# Interaction of KSHV with Host Cell Surface Receptors and Cell Entry

**DOI:** 10.3390/v6104024

**Published:** 2014-10-23

**Authors:** Mohanan Valiya Veettil, Chirosree Bandyopadhyay, Dipanjan Dutta, Bala Chandran

**Affiliations:** H. M. Bligh Cancer Research Laboratories, Department of Microbiology and Immunology, Chicago Medical School, Rosalind Franklin University of Medicine and Science, North Chicago, IL 60064, USA; E-Mails: chirosree.bandyopadhyay@my.rfums.org (C.B.); dipanjan.dutta@rosalindfranklin.edu (D.D.); bala.chandran@rosalindfranklin.edu (B.C.)

**Keywords:** KSHV, HHV-8, receptors, integrins, signal induction, mode of entry

## Abstract

Virus entry is a complex process characterized by a sequence of events. Since the discovery of KSHV in 1994, tremendous progress has been made in our understanding of KSHV entry into its* in vitro* target cells. KSHV entry is a complex multistep process involving viral envelope glycoproteins and several cell surface molecules that is utilized by KSHV for its attachment and entry. KSHV has a broad cell tropism and the attachment and receptor engagement on target cells have an important role in determining the cell type-specific mode of entry. KSHV utilizes heparan sulfate, integrins and EphrinA2 molecules as receptors which results in the activation of host cell pre-existing signal pathways that facilitate the subsequent cascade of events resulting in the rapid entry of virus particles, trafficking towards the nucleus followed by viral and host gene expression. KSHV enters human fibroblast cells by dynamin dependant clathrin mediated endocytosis and by dynamin independent macropinocytosis in dermal endothelial cells. Once internalized into endosomes, fusion of the viral envelope with the endosomal membranes in an acidification dependent manner results in the release of capsids which subsequently reaches the nuclear pore vicinity leading to the delivery of viral DNA into the nucleus. In this review, we discuss the principal mechanisms that enable KSHV to interact with the host cell surface receptors as well as the mechanisms that are required to modulate cell signaling machinery for a successful entry.

## 1. Introduction

Herpesviruses have evolved to engage multiple host cell plasma membrane molecules to penetrate the target cells first line of defense [1]. Some of these molecules are utilized as “binding receptors” which enable the virus to initially attach and concentrate on the surface of the cells while others designated as “entry receptors” are utilized to trigger either the fusion of viral envelope with the plasma membrane in neutral pH or entry of the whole virus particle by endocytosis and subsequent fusion of viral envelope with the endosome membranes in an acidic or non-acidic environment [1,2]. Upon successful penetration of the plasma membrane barrier, herpesviruses employ different intracellular organelles and cytoskeletal routes to migrate towards the nucleus, disassembly of capsid near the nuclear pore, with subsequent delivery of genome into the nucleus and infection leading into production of progeny virus and/or establishment of a characteristic lifelong latent infection in cell type specific manner [1,2].

Kaposi’s sarcoma associated herpes virus (KSHV) or human herpesvirus-8 (HHV-8), discovered in 1994, is classified as a member of the γ2-lymphotropic-ongogenic herpesviruses [3,4,5]. KSHV is etiologically associated with Kaposi’s sarcoma (KS) and with two lymphoproliferative malignancies, primary effusion lymphoma (PEL) and multicentric Castleman’s disease (MCD) [6,7,8]. The KSHV genome is closely aligned with γ-1 Epstein-Barr virus (EBV) and with γ-2 herpesvirus saimiri (HVS) and Rhesus monkey rhadinovirus (RRV). Similar to all herpesvirus family members, KSHV has a double stranded DNA genome (~160 kb) packed inside a capsid surrounded by a tegument which is further enclosed by a lipid envelope with five conserved glycoproteins [9,10]. The KSHV genome encodes more than 100 open reading frames (ORFs), of which 4 to 75 are classified by their homology to HVS ORFs [11]. The genome consists of conserved gene blocks overlapping with other herpesvirus family members, as well as >20 genes (K genes) unique to KSHV.

KSHV displays a broad cellular tropism as it infects a variety of target cells* in vitro* and* in vivo* [12]. KSHV entry and signal induction is a complex event and greatly varies according to cellular tropism [13]. KSHV utilizes different combinations of host cell surface receptors, and targets different internalization pathways by selectively inducing specific downstream signal molecules [13]. Independent studies have shown that multiple KSHV glycoproteins engaging host cell membrane binding and entry receptors induce cascades of signal pathways promoting endocytosis. Subsequent steps include fusion of the viral envelope with endosomal membranes, release of virus capsid in the cytosol, capsid trafficking to the nuclear periphery, and delivery of KSHV DNA into the nucleus [13]. Therefore, these overlapping phases are essential for KSHV *de novo* infection, which relies on intricate spatio-temporal dynamics of molecular interplay.

This review summarizes almost two decades of extensive research findings by several groups regarding KSHV receptors, entry pathways, trafficking and early immune modulation during *de novo* infection of target cells. While advances have been made in our understanding of the entry associated signaling events early during KSHV-cell interaction, information regarding KSHV trafficking and nuclear entry remains incomplete. Hence, this review also highlights current perspectiveson KSHV early events that several groups have reported over the decades of research in the field of KSHV biology.

## 2. KSHV Envelope Glycoproteins

The envelope glycoproteins of KSHV play an important role in infection as they mediate virus-cell initial attachment, entry, assembly, and egress of the virus. KSHV ORFs 8, 22, 47, 39, and 53 encode envelope glycoproteins gB, gH, gL, gM, and gN, respectively, which are conserved among other herpesviruses [4,12,14]. KSHV also encodes unique lytic cycle associated glycoproteins ORF4, gpK8.1A, gpK8.1B, K1, K14, and K15 [4,12,14], with ORF4 and gpK8.1A as part of the envelope of KSHV [15,16,17,18,19,20,21,22].

KSHV gB is a key envelope glycoprotein involved in the initiation of entry. gB is synthesized in a precursor form as a 110-kDa polypeptide which is further proteolytically cleaved and processed to produce disulfide linked mature polypeptides of molecular weight 75 and 54-kDa [15,17,23]. gB imparts a major functionality in primary virus-cell interaction by binding to cell surface binding receptor heparan sulfate, and entry receptors α3β1, αVβ3, and αVβ5 integrins [17,24,25]. gB has also been shown to bind to the DC-SIGN receptor [26]. The interaction of KSHV gB with host cell surface receptors activates the host’s integrin associated pre-existing signal molecules such as FAK, Src, PI3-K, and Rho-GTPase [27].

Unlike other herpesviruses, lytic phase associated glycoproteins gpK8.1A and gpK8.1B are produced from alternatively spliced messages of the gpK8.1 gene. gpK8.1A is the main form expressed in infected cells and assembled in the virion envelope [4,28,29]. Functionally both gB and gpK8.1A interact with KSHV binding receptor HS [20], and are also enriched in the membrane lipid raft microdomains of infected endothelial cells [30,31].

Similar to other herpesviruses, KSHV glycoproteins gH and gL form a non-covalently linked gH/gL complex, where 120-kDa gH combines with 42-kDa gL. gL plays a lead role in gH/gL complex formation by promoting intracellular gH trafficking [21]. gH and complement binding ORF4 are shown to interact with heparan sulfate [32,33], whereas, studies have also demonstrated that gH/gL antibody treatment affects KSHV entry without affecting KSHV binding [21]. Recently, gH/gL were demonstrated to interact with KSHV entry receptor EphA2 and are indispensable for KSHV entry [33].

KSHV glycoproteins gM and gN are N-glycosylated to form a heterodimeric complex and functionally participate in virus penetration and egress. Like gH/gL synergy, gN has been reported to be required for post-translational modification of gM and trafficking to the cell surface [22].

## 3. Target Cell Receptors for KSHV Entry

Like α, β, and γ-herpesviruses, KSHV broadly recognizes two categories of cellular receptors, binding and entry receptors. The binding receptor for KSHV is host cell surface heparan sulfate (HS) which promotes a charge based interaction between virus glycoproteins and cellular HS, enabling KSHV to attach and concentrate on target cells. The entry receptors of KSHV are highly specific and utilized in different combinations which greatly vary according to cellular tropism as well as the entry pathways exploited by the virus. Entry receptor utilization is also a primary step in routing KSHV containing cargo to productive *vs* non-productive pathways of infection [31].

### 3.1. Heparan Sulfate as Binding Receptors for KSHV

Heparan sulfates are ubiquitously expressed cell membrane proteoglycans with charged carbohydrate moieties that interact with several protein ligands and extensively studied in herpesviruses. HS are known to facilitate KSHV attachment and concentration on the cell surface, enabling possible conformational change(s) in virus glycoprotein(s) to gain access to specific adjacent entry receptors [17]. This initial attachment step by a universal receptor partly explains the broad cellular tropism for KSHV. Pretreatment of KSHV with soluble heparin results in dose dependent inhibition of KSHV binding and subsequently virus induced signal induction [17]. However, soluble chondroitin sulfate A and C treatment did not prevent KSHV infection, demonstrating the specificity of HS in KSHV-cell interaction [17]. Involvement of HS in influencing KSHV cellular infectivity is also examined in several B cell lines and primary B cells defective for HS biosynthesis. B cells and cell lines lacking Ext1 enzyme are unable to promote the crucial glycosylation step in HS biosynthesis, and lower expression of HS limits KSHV infectivity in these cells. Studies with BJAB (KSHV and EBV negative B-cell lymphoma line) cells support the notion as expression of HS in those cells enhance the susceptibility of KSHV infection, whereas KSHV fails to infect BJAB cells lacking HS expression [34].

KSHV utilizes several of its envelope glycoproteins such as gB, gpK8.1A, ORF4, and gH for its binding to cell surface HS molecules which probably facilitates speedy concentration of virus particles on the cell surface in an extracellular environment of rapid fluid movement [19,23,25,33]. Several blocking approaches taken towards target cell surface HS also indicates the necessity of HS for KSHV infection. For example, pretreatment of target cells either with enzyme heparinase I and III to cleave surface HS or pretreatment with a soluble form of KSHV gB and gpK8.1A to saturate surface HS binding, can successfully block KSHV infectivity [19,20,23].

Biochemical characterization demonstrating the presence of heparin binding domains (HBD) provides evidence that the KSHV gB extracellular domain possesses a conserved motif HIFKVRRYRK (108–117) and KSHV gpK8.1A has atypical HBDs whereas gH lacks it [17,21]. Recombinant purified forms of KSHV envelope glycoproteins gB and gpK8.1A can bind specifically heparin-agarose and saturate target cell surface HS molecules without any affinity to chondroitin sulfates [20,23].

### 3.2. Integrins as Entry Receptors for KSHV

Integrins are extracellular cell surface receptors, well known for major extracellular matrix (ECM) outside-in signaling. KSHV was the first herpesvirus shown to utilize integrins as entry receptors in adherent target cells [25]. Several lines of evidence demonstrate that α3β1, αVβ3, and αVβ5 integrins play a crucial role in KSHV infection [24,25,35,36]. Like many ECM proteins, KSHV gB possesses a traditional integrin binding Arg-Gly-Asp (RGD) motif at amino acids 27–29 [25]. Pretreatment of HMVEC-d (human microvascular dermal endothelial cells) and HFF cells (human foreskin fibroblasts) with soluble RGD peptides, antibodies against RGD-gB peptides (RGDTFQTSSSPTPPGSSS), and fibronectins have shown the necessity of integrins as entry receptors for KSHV target cell infection [25]. Moreover, anti-αV and anti-β1 integrin antibody treatment inhibited KSHV-gB mediated cell attachment in HMVEC-d, HFF-cells, monkey kidney cell line CV-1, and human fibrosarcoma cell line HT-1080 [24,25,36]. Functional blocking of α3β1 integrin shows 30%–50% reduction in KSHV infection whereas overexpression of α3 integrin in Chinese hamster ovary (CHO) cells that eventually forms a complex with hamster β1 integrin increasing KSHV infectivity [25]. However, α3 over expression does not attain the level of KSHV infection observed in HMVEC-d and HFF cells that justifies the need for multiple receptor engagement by KSHV [24]. Studies involving various cell types such as HEK293, HMVEC-d, HFF, and Vero cells, show that pretreatment with soluble α3β1, αVβ3, and αVβ5 integrins inhibit KSHV infection [24]. Microscopic evidences also support that integrins form a multimolecular receptor complex during KSHV entry into target cells [24]. The role of integrins in KSHV entry is also characterized in monocytes [35].

There are some discrepancies regarding the role of integrin subtype used by KSHV in different target cells [36,37]. However, experimental methodologies utilized in those studies explain the reason behind dissimilar findings. For example, one study was unable to observe the ability of soluble α3β1 and RGD peptides to block KSHV infectivity in the 293-T cell line used a different infection method [37]. In this study, the cells were infected with KSHV by using centrifugation and polybrene. This delivery methodology tools enhances infectivity for any virus infection as it bypasses the requirement of virus attachment with specific host cell surface receptors. In fact, polybrene is classically used as a gene delivery agent for many viruses as it effectively coats the viral envelope and increases target cell transduction.

Another study, utilizing a 15-mer-AHSRGDTFQTSSGCG peptide of KSHV-gB, demonstrated that this peptide mediated cell adhesion in fibrosarcoma HT1080 blocked by αVβ3 antibodies, but not by α3 and α5β1 antibodies. This study showed that αVβ3 mediates both the cell adhesion and entry of KSHV into target cells through interaction with the RGD motif of gB [36]. However, KSHV gB lacks the GCG amino acid peptide used in the study and may form dimers and multimers due to the cysteine residues [36]. The ability of anti- α3β1 and anti- αVβ5 antibodies to block KSHV infection was not tested in this study to conclusively demonstrate that αVβ3 integrin functions as entry receptors for KSHV in HT1080 cells [36]. Moreover, the choice of target cell is not appropriate since HT1080 susceptibility towards KSHV infection remains a concern [24].

Mouse keratinocytes, negative for α3β1, are KSHV susceptible and α3 overexpression resulted in a 55% reduction of infection in these cells [36]. Even though the cell surface expression and availability of other integrins such as αVβ3 and αVβ5 or experiments with function blocking antibodies were not tested in those cells, it was concluded that α3β1 expression has a dominant effect on αvβ3 expression. This study also does not provide experimental justification as why α3β1 overexpression may have a dominant negative effect on αVβ3. Therefore, it remains a concern why virus binding to α3β1 integrin would generate a dominant negative effect on another cell surface molecule and would be of great interest to elucidate further.

Later studies highlight the cell surface lipid raft* vs.* non-lipid raft localization of integrin receptors as an important criterion for KSHV infection of target cells [31]. Studies also clearly demonstrate that it’s not a single entry receptor and virus glycoprotein interaction event but virus-host cell surface interaction followed by consequent conformational changes in the cell surface receptor that regulate multi-molecular complex formation and subsequent stages towards a successful infection [24,31].

### 3.3. Potential participation of gB Disintegrin in KSHV Entry

Recent studies indicate that KSHV gB also contain an integrin-binding disintegrin-like domain, a sequence similar to the RX_6–8_DLXXF found in the ADAM family of proteins [38]. By using phage display peptide library screening, it was identified that the disintegrin-like domain DLD of gB interacts with α9β1 integrin receptor and lead to binding and virus entry. Treatment of HFF and HMVEC-d cells with the function blocking α9 and β1 integrin antibodies, anti-DLD antibodies to the disintegrin domain, and soluble integrin treated KSHV resulted in reduced binding and entry of KSHV into the target cells [38]. These results therefore establish the importance of the disintegrin-like domain in KSHV entry and show that α9β1 is a gB disintegrin domain binding receptor for KSHV. However these studies did not examine whether the β1antibodies also block α3β1 integrin and the role of α9β1 integrin in infectivity in the context of α3β1, αVβ3 and αVβ5 receptors is not clear.

### 3.4. xCT as Entry Receptor for KSHV

xCT, a 12-transmembrane glutamate/cystine exchange transporter was identified in 2006 as a fusion-entry receptor for KSHV in target cells [39]. xCT forms a complex with cell surface 125-kDa disulfide linked heterodimeric membrane glycoprotein CD98 (4F2 antigen) which contains a common glycosylated heavy chain (80-kDa) and a group of 45-kDa light chains where xCT is a light chain [40,41,42]. CD98, originally identified as integrin α3 associated molecule, regulates amino-acid transport, cell adhesion, fusion, proliferation, and integrin activation. CD98 and integrin α3 were later identified as fusion regulating protein 1 (FRP-1) and FRP-2, respectively, in mediating cell-cell fusion and virus-induced cell fusion [40,41,42]. Further studies identified xCT as a member of a multimolecular signaling complex assembled during KSHV macropinocytosis in HMVEC-d cells [24,31]. It is likely that xCT can direct a specific kind of downstream signaling to facilitate KSHV endocytic events and interaction of xCT with multiple integrin receptors promoting signal clustering can potentiate specific functions although additional investigation is required. However, it is shown that heparin and soluble α3β1 integrin pretreatment inhibits α3β1-CD98/xCT complex formation which advocates for KSHV’s initial binding with HS, followed by integrin interaction, possible conformational changes in envelope glycoproteins leading to CD98/xCT association [24]. Hence, it would be highly interesting to delineate the specific KSHV glycoprotein responsible for xCT interaction.

The interaction of KSHV receptors and rapid multimerization of receptors occurs within 1 min post-infection [24]. The formation of such a multimolecular receptor complex at the membrane could be an essential step in coordinating the intricate molecular and cellular set-up of the target cells to allow entry of the virus. Since heparin treated virus blocked the multimerization process, it was suggested that binding of KSHV to HS is the primary signal which leads to the induction of receptor and ultimately results in multimolecular complex formation and endocytosis of KSHV [24]. Results from several other laboratories also suggest that the HS must be exposed on the cell surface for binding and the subsequent interaction with receptors to promote internalization and a productive infection of the virus [19,20,23,25].

In recent studies using DNP labeled KSHV virions and tyramide signal amplification (TSA) confocal microscopy, Garrigues et al confirmed KSHV-dependent clustering of integrins α3β1, αVβ3, αVβ5 and CD98 in HT1080 cells [43]. However, this study made contradictory observations with respect to receptor localization at KSHV bound cell surface microdomains. According to this study, the initial binding of KSHV across the cell membrane is not dependent on HS, but it utilizes αVβ3 for initial attachment. The relationships between KSHV infectivity and αVβ3 receptor expression were also analyzed in human salivary gland epithelial (HSG) cells that lack αVβ3 but express high levels of heparan sulphate and other KSHV receptors. These cell lines were resistant to KSHV infection, but reconstitution of αVβ3 receptor enhanced KSHV entry [43]. From this study, it was concluded that the αVβ3 has a significant impact on the initial binding of KSHV to the cell surface and changes the infectivity of cells. Quantitative analysis is important to understand the relative changes in viral gene copy numbers in the infected cells. However, these experiments used immunofluorescence analysis to detect the number of infected cells. Another notable weakness of this study is that the infection was analyzed by the expression of LANA at a later time point of infection, which is not a standard assay to determine the sequential role of receptors in binding and entry. This study also lacks experiments to determine whether treatment of the virus with heparin inhibits KSHV entry in αVβ3 expressing cells. Therefore, the conclusion that the initial contact with the αVβ3 receptor allows the virus to trigger the entry mediated events is uncertain although αVβ3 has been shown to be a part of the multimolecular complex and utilize αVβ3 as a functional receptor to mediate KSHV entry.

### 3.5. DC-SIGN as Entry Receptor for KSHV

Dendritic cell specific intracellular adhesion molecule-3 (ICAM-3) grabbing non-integrin (DC-SIGN) is a C-type lectin typically expressed on the DC cell surface and known to be utilized by many viruses as a receptor including human immunodeficiency virus (HIV) and Bunyaviruses [44,45]. Likewise, KSHV also exploits DC-SIGN during infection of human myeloid dendritic cells (DCs), macrophages, and activated B cells [46,47]. Pretreatment with anti-DC-SIGN monoclonal antibody, mannan (a natural ligand of DC-SIGN), and soluble DC-SIGN is shown to inhibit KSHV binding and infection [46,47]. Moreover, recent studies have shown B cells are more susceptible towards KSHV infection due to increased expression of DC-SIGN. This reflects a role for DC-SIGN in mediating the entry process of KSHV. However, a partial block in KSHV infection upon anti-DC-SIGN monoclonal antibody treatment explains the need for additional binding receptors such as HS and/or other co‑receptors in cell types that utilize DC-SIGN as a receptor for entry. KSHV gB which has abundant mannose sugar residues is reported to bind with DC-SIGN and promote the entry of KSHV [26]. KSHV is capable of using HS and integrins to bind and effectively enter into THP-1 cells. Interestingly, DC-SIGN is reported as an entry receptor for KSHV in THP-1 cells in addition to previously identified integrins since functional blocking of DC-SIGN affects KSHV entry but not binding in those cells [35].

### 3.6. EphA2 as Entry Receptor for KSHV

Ephrin receptors, the largest family of tyrosine kinase receptors, are known to mediate diverse activities such as integrin associated signaling, actin cytoskeleton assembly, cell adhesion, and cell movement, with implications in neovascularization and oncogenesis. Ephrins have been implicated as a hub for signaling events and ephrin receptors control macropinocytosis and clathrin dependant endocytosis in various cell types [48,49]. A recent report suggests that the interaction of ephrin receptor A2 (EphA2) tyrosine kinase with the KSHV glycoproteins gH and gL results in entry of the virus. Pretreatment of the target cells with a soluble EphA2 ligand or preincubatiion of KSHV virions with soluble EphA2 inhibited KSHV infection. The specific role of EphA2 in KSHV entry was also established using an EphA2 knockdown and overexpression system. EphA2 knockdown correlated with a significant decrease in KSHV entry, whereas overexpression of EphA2 increased viral entry. Furthermore, gH/gL binding with EphA2 induced EphA2 phosphorylation and internalization of the virus. These findings suggest that EphA2 is a specific cellular receptor for KSHV (Table 1) [50].

**Table 1:** Binding and entry receptors of KSHV in various target cells.

Target Cells	Binding Receptors	Entry Receptors
Human foreskin fibroblasts (HFF)	HS	α3β1, αVβ3, αVβ5, xCT/CD98, EphA2 [24,25,38,39,51]
Human microvascular dermal endothelial cells (HMVEC-d)	HS	α3β1, αVβ3, αVβ5, xCT/CD98, EphA2 [24,25,38,39,50,52]
Human embryonic kidney epithelial cells (HEK293)	HS	α3β1, αVβ3, αVβ5, xCT/CD98 [24]
Monocytes	HS, DC-SIGN	DC-SIGN, α3β1, αVβ3, αVβ5 [35]
B cells, macrophages, dendritic cells	DC-SIGN, ?	DC-SIGN [46,47], ?

**Figure 1:**
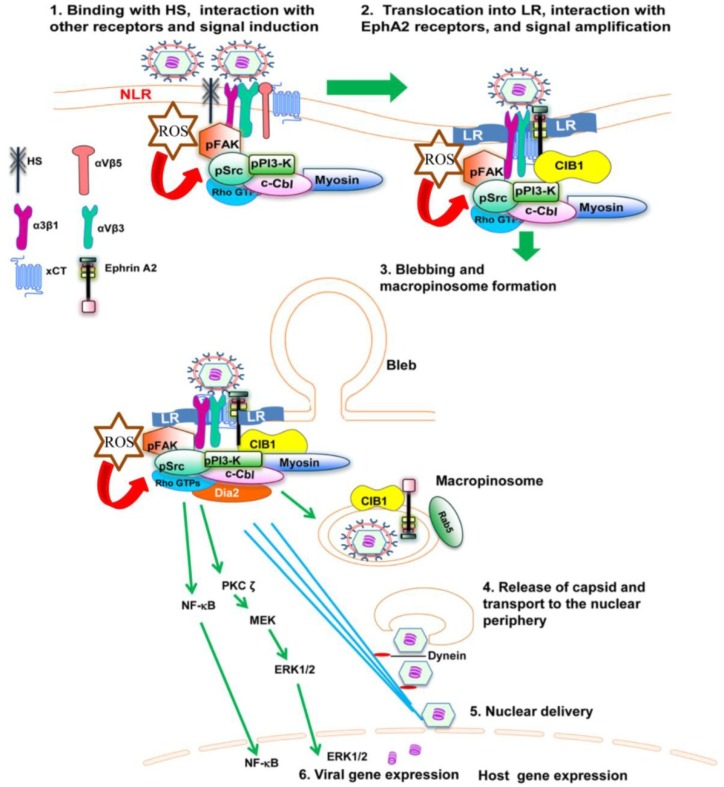
Diagram depicting the sequence of events in macropinocytosis of KSHV in human microvascular dermal endothelial cells. (**1**) The initial attachment of KSHV with HS is followed by interaction with α3β1, αVβ3 and αVβ5 integrins, EphA2 and xCT molecules in the non-lipid raft (NLR) region of the membranes; (**2**) The interaction of KSHV with receptors induces the phosphorylation of FAK, Src, PI3-K as well as recruitment of the adaptor proteins CIB1, c-Cbl and rapid translocation of KSHV into the LR along with the α3β1, αVβ3, and x-CT receptors but not αVβ5. The interaction of KSHV with receptors also induces the production of ROS, which in turn stimulate the signaling molecules FAK, Src and the Rho GTPase Rac1; (**3**) The activated c-Cbl interacts with myosin IIA and results in bleb formation, bleb retraction and macropinosome formation along with the viral particles, EphA2 and CIB1. Rab5 is also recruited to the internalized macropinosome membrane; (**4**) The endosomal membranes fuse with the viral glycoproteins and release the KSHV capsid into the cytoplasm. RhoA GTPase mediated Dia-2 induces the acetylation of microtubules, and helps the transport of capsid towards the nucleus; (**5**) Capsid disassembly and delivery of viral DNA into the nucleus; (**6**) Viral gene expression occurs with the help of host signaling molecules ERK and NF-κB.

Studies supporting the importance of EphA2 for KSHV infection showed that EphA2 shRNA, monoclonal antibodies, and tyrosine kinase inhibitor blocked macropinocytosis of KSHV into dermal endothelial cells. KSHV’s binding and interaction with heparan sulphate, integrins (α3β1, αVβ3, αVβ5) and x-CT initially occurs in non-LR (non-lipid raft) regions. The association of KSHV with receptors is followed by c-Cbl–mediated rapid translocation of KSHV along with selective integrins (α3β1, αVβ3) and x-CT receptors into LRs (lipid raft) [52]. Integrin translocation into the LR promotes the association with EphA2, which in turn results in the formation of an active signalling complex between integrins, c-Cbl and myosin IIA, thereby inducing macropinocytic blebs. EphA2 also binds several signaling molecules, including FAK, Src, and c-Cbl-myosin IIA complex in the LRs [52]. The formation of such signaling complexes allows the retraction of blebs and macropinocytosis of KSHV into early macropinosomes. The macropinosomes move toward the nucleus resulting in nuclear delivery and gene expression (Figure 1). Another study demonstrates that EphA2 plays a crucial role in coordinating and amplifying KSHV induced signaling essential for virus internalization through clathrin mediated endocytosis (CME) in human fibroblast cells [51].

## 4. KSHV Induced Signal Pathways

The interactions of KSHV glycoproteins with the integrins and other cellular receptors trigger the induction of intracellular tyrosine kinases, and organization of the actin cytoskeleton. Integrins directly induce autophosphorylation of FAK and the phosphorylated FAK interacts with downstream effectors to modulate various aspects of infection. The majority of downstream mediators, such as Src, PI3-K, and c-Cbl, possess characteristic SH2 and SH3 domains or binding sites for those domain containing proteins [53], which potentiate subsequent coupling and signal transduction cascades induced by KSHV. They facilitate KSHV entry by protein-protein interaction and by enzymatic (kinase and ubiquitin ligase) action [31,54]. Many of these signaling molecules are critical determinants in KSHV entry.

### 4.1. Role of FAK and Src in KSHV Entry

Focal adhesion kinase (FAK) is a critical tyrosine kinase activated by integrins and is involved in multiple biological functions, including cell adhesion, proliferation, migration, and endocytosis [55,56]. Activation of the FAK signaling cascade in KSHV infected HMVEC-d, HFF, HEK293, and FAK +/+ mouse Du17 fibroblasts promotes entry and subsequent steps of infection [23,25,27,57,58,59]. The FAK dependent signaling cascade in the infected cells is initiated by the autophosphorylation of FAK at tyrosine 397, which is a major phosphorylation site required for the outside-in signaling of integrins [55,60]. Phosphorylated FAK assembles a membrane bound signaling complex and also links other kinases to downstream signaling events, thereby allowing entry of the virus into the cells. FAK positive mouse fibroblasts reduced the entry of KSHV, whereas the FAK negative mutant did not show any decrease in the entry of KSHV, indicating that phosphorylation of FAK and FAK induced signaling is important for entry of the virus [57,61]. KSHV soluble glycoprotein gB is also known to elicit phosphorylation of FAK [23,27].

Phosphorylated FAK associates with Src, RhoA, and cytoskeletal proteins like vinculin and paxillin in the infected cells [23,25,27,58]. Studies supporting the importance of this association for KSHV infection showed that these molecules enhance the signals generated by FAK and regulate entry of the virus. Phosphorylated Src colocalizes with FAK and induces a variety of intracellular signaling by phosphorylating PI3-K and other downstream targets such as Rho-GTPases [58]. Moreover, Src kinases are also important for the endocytosis of KSHV. The finding that Src kinase activity and KSHV entry can be increased by LR disruption suggests that Src recruitment to the LR compartment is required for the regulated entry of KSHV in target cells [59]. The association of RhoA with Src and feedback activation of Src is required for the internalization of KSHV in HEK293 cells [58]. The coordinated activities of these proteins play a significant role in regulating the mechanism of KSHV entry and trafficking.

### 4.2. PI3-K and RhoA-GTPase Crosstalk in KSHV Entry and Nuclear Delivery

PI3-K is activated downstream to integrin and receptor tyrosine kinase (RTK) pathways upon specific tyrosine phosphorylation of the p85 regulatory subunit and activity coordinated by the p110 catalytic subunit. PI3-K critically transmits signals through several signal pathways such as activating Rho-GTPase, apoptosis, survival, and migration [55,62]. PI3-K is implicated as a KSHV entry associated signal mediator. KSHV induces PI3-K tyrosine phosphorylation as early as 5 min p.i., which decreases after 15 min [57]. KSHV induced PI3-K activation is inhibited by selective inhibitors such as wortmannin and LY294002 in a dose dependent manner that functionally inhibited KSHV entry [57]. Binding deficient heparin treated KSHV is unable to induce PI3-K phosphorylation and inhibition of Src kinases by SU6656 also prevents KSHV-gB induced PI3-K p85 phosphorylation [27]. Further examination of PI3-K upstream pathways early during KSHV entry in FAK positive Du17 cells and FAK negative Du3 cells supports the notion that integrin associated FAK activation is absolutely necessary for KSHV gB mediated PI3-K activation [27]. KSHV entry receptor EphA2 also mediated PI3-K signal augmentation and recruitment to the entry associated signal complex in HMVEC-d cell lipid raft and HFF cell non-lipid raft regions [51,52]. PI3-K activation sends signals to downstream RhoA GTPases and additional effectors to promote different stages of endosome formation and endosome trafficking during KSHV entry [58].

RhoA GTPase family members RhoA, Rac, and Cdc42 are involved in a variety of cellular signaling pathways including cytoskeletal rearrangement and morphological changes [55,63,64]. KSHV induced cytoskeletal rearrangement is PI3-K Rho-GTPase dependent and triggers lamellipodia (Rac), filopodia (Cdc42), and stress fibre (RhoA) formation [57,58,65,66]. Recombinant KSHV gB induces RhoA GTPase signaling through activation of upstream FAK-Src-PI3-K signal pathways [27]. In addition, the actin cross-linking molecule ezrin participates in events downstream to Rho-GTPase signaling [27]. Studies using RhoA inhibitor *Clostridium difficile* toxin B (CdTxB), and overexpression of dominant-negative RhoA demonstrate significant reduction in KSHV entry [58].

RhoA GTPase in regulating downstream formin family members diaphanous 1 and 2 (Dia-1 and 2) was demonstrated by several studies [63,64,67]. KSHV infection also induces Dia-2 as a RhoA downstream event without any significant induction in Rac-1 and Cdc42 mediated PAK1/2 or stathmin molecules [68]. Dia-2 associates with activated Src and Src inhibitor affects Dia-2 action. Indeed, functionality of KSHV induced RhoA and Dia-2 signaling is coupled as a probable link between RhoA and sustained feedback activation of Src [58]. These studies suggest that RhoA GTPase pathway is an important signaling pathway that regulates endocytosis of KSHV.

Recent findings indicate that reactive oxygen species (ROS) generated by KSHV also plays an important role in the entry of the virus. Treatment with the ROS inhibitor N-acetyl cysteine (NAC) reduced KSHV infection by blocking virus entry, membrane bleb formation, and the phosphorylation of the ephrin-A2 receptor, FAK, Src, and Rac1 [69]. These studies demonstrate KSHV induced ROS promote KSHV entry and the amplification of the initial host signal cascade, including EphA2, FAK, Src, and the Rho GTPase Rac1 (Figure 1).

### 4.3. c-Cbl in Adapting KSHV Entry

Classically, proteins possessing characteristic domains such as SH2 and SH3 (SH = Src homology) domains, PDZ domain etc, or such domain binding motifs that lack enzymatic activity and mediate protein-protein interaction are defined as adaptor molecules. [70]. c-Cbl is a multifunctional adaptor protein capable of communicating between a plethora of signal pathways. c-Cbl being an E3 ubiquitin ligase influences cellular signal pathways by ubiquitinating target proteins to control their localization, phosphorylation, and interaction with other signaling partners [71,72].

The adaptor function of c-Cbl is extensively studied in KSHV *de novo* infection [31,54]. KSHV induced c-Cbl tyrosine phosphorylation occurs as early as 1 min p.i., is required for the formation of spherical membrane protrusions called blebs to promote bleb mediated macropinocytosis of KSHV [54]. c-Cbl induction by KSHV is downstream to PI3-K and c-Cbl recruitment to the junctional bases of macropinocytic blebs is dependent on its novel interacting partner myosin IIA very early at 5 min p.i. [54]. c-Cbl shRNA transduced HMVEC-d cells inhibited KSHV macropinocytosis as well as KSHV gene expression. The role of c-Cbl in promoting macropinocytosis is reported for the first time in KSHV macropinocytosis [54]. Taken together, this study demonstrates the absolute requirement of the c-Cbl-myosin IIA interaction and c-Cbl mediated myosin IIA ubiquitination in bleb mediated macropinocytosis of KSHV in HMVEC-d cells (Figure 1).

Simultaneously, enzymatic action of c-Cbl (*i.e.*, ubiquitination) is differentially employed by KSHV in HMVEC-d cells to dictate KSHV bound entry receptor internalization pathways and consequently the fate of the virus [31]. Independent studies report that c-Cbl is capable of ubiquitinating KSHV entry receptor β1 integrins and initiating virus internalization in HMVEC-d and HUVEC cells [31,73]. The study in HUVEC cells observed that c-Cbl favors KSHV clathrin mediated internalization via β1 integrin ubiquitination, however, the reported c-Cbl functionality is not consistent with the mechanism published by another group [31]. These dissimilar findings in HUVEC cells can be explained as the study monitors KSHV entry associated events in HUVEC cells at 1 hour post-infection or at even later time points, 4 and 8 h.

In contrast, parallel studies on KSHV entry pathways in dermal endothelial cells report that c-Cbl selectively monoubiquitinates KSHV entry receptors integrin β1 and β3 molecules to facilitate KSHV macropinocytosis leading towards a successful infection whereas c-Cbl polyubiquitinates integrin β5 to direct clathrin mediated KSHV endocytosis and for directing KSHV towards lysosomal degradative pathways [31]. Hence, the mechanistic purpose of such differential ubiquitination is a key receptor associated signaling event aiding KSHV to utilize particular cellular entry pathways and manipulate the event for its own benefit.

KSHV utilizes the same adaptor function of c-Cbl in HFF cells to facilitate clathrin-mediated endocytosis [51]. In HFF cells, KSHV infection engages c-Cbl with its tyrosine kinase binding (TKB) and RING domains with tyrosine kinase (TK) and sterile alpha motif (SAM) domains of EphA2, to facilitate polyubiquitination (K63 type) of the EphA2 receptor to promote clathrin mediated internalization of associated virus [51]. c-Cbl siRNA studies in HFF cells, inhibit KSHV association with clathrin and EphA2 receptor [51]. These studies demonstrate that KSHV has evolved to exploit c-Cbl function selectively to display its broad cellular tropism.

### 4.4. CIB1 Mimics Adaptor Function during KSHV Entry

A recent report identifies CIB1 (Calcium and integrin binding protein-1) as a novel macropinosome associated molecule exerting adaptor function to promote KSHV macropinocytosis in endothelial cells [74]. CIB1, a ubiquitously expressed 22-kDa calcium binding protein, originally identified as an αIIβ3 integrin binding protein in platelets, is involved in regulating cell spread and motility. Structurally CIB1 is an EF hand (basic helix-loop-loop-helix) family protein, in particular, it is a compact alpha helical protein with four EF hands which lacks any anti-parallel beta sheets required to form traditional SH2 (a central anti-parallel beta sheet surrounded by two alpha helices) and SH3 domains (five anti parallel beta strands packed perpendicularly to two perpendicular beta sheets) [75,76].

CIB1 is reported as a key effector molecule promoting EphA2 associated signal events during KSHV entry [74]. CIB1 knockdown studies demonstrate significant reduction in KSHV-induced bleb formation, activation of EphA2, Src, and Erk1/2, virus entry by macropinocytosis, productive trafficking, and infection [74]. Studies also report CIB1 playing a role in scaffolding EphA2 with cytoskeletal myosin IIA and alpha-actinin 4 during KSHV entry [74]. Simultaneously, overexpression of CIB1 in HEK293 cells has the potential to increase KSHV entry by 60% which correlates with the finding of a reduction in KSHV entry by ~68% in CIB1 knockdown HMVEC-d cells. Together, these studies reveal for the first time the role of CIB1 as a potential adaptor molecule in virus macropinocytic entry and promote CIB1 as an attractive target to block KSHV entry and infection. While the experiments with purified recombinant KSHV gB sheds light on a probable role of gB in recruiting CIB1 to the KSHV induced membrane blebs, the direct recruiting partner of CIB1 to the KSHV induced signal complex remains uncharacterized.

KSHV is known to induce calcium immediately (~30 seconds) after infection in HUVEC cells via Src induction and Src association with plasma membrane associated L-type calcium channel Cav1.2 [77]. However, the study focused on the role of calcium in mobilization of cytokine stores such as Angiopoietin-2 secretion. Calcium is a highly important divalent cation that regulates several signaling events and in cellular motility actions, namely, in membrane blebbing, integrin signaling, vesicular trafficking, *etc*. [78]. Moreover, calcium plays an important role in Herpes simplex virus and Coxsackie bar virus entry associated signaling [79,80]. Hence, it would be of great interest to test any potential role of calcium influx in CIB1 mediated cellular signaling during KSHV entry.

## 5. Role of Lipid Rafts in KSHV Entry

By definition, lipid rafts (LR) are cholesterol and sphingolipid enriched, detergent resistant, floating microdomains in the exoplasmic leaflet of plasma membranes involved in major outside-in signaling events via promoting receptor clustering, protein-protein and protein-lipid interactions [81]. Lipid rafts are known to play a critical role in KSHV *de novo* infection [59]. Earlier reports on KSHV entry demonstrate that LR disrupting agents such as nystatin or methyl beta cyclodextrin have no effect on KSHV binding, increase KSHV entry but decrease virus association with microtubules due to microtubule disorganization and consequently decrease KSHV nuclear delivery [59]. Subsequent studies report the complex regulation of KSHV induced signals by LRs. For example, LR disruption increases p-Src induction by KSHV without affecting FAK or ERK1/2 activation but greatly reduces PI3-K, Rho-GTPase, and NF-κB activation. Consequently, RhoA mediated acetylation and microtubule aggregation also gets abolished [59].

The mechanistic role of LRs is thoroughly studied during KSHV entry in HMVEC-d cells [31]. Early during KSHV infection, HMVEC-d cell LRs act as a signaling hub to promote virus and multiple receptor clustering and are also capable of recruiting key cytosolic entry mediators such as c-Cbl [31]. KSHV induced c-Cbl monoubiquitinates α3β1 and αVβ3 integrins resulting in the rapid lateral translocation of virus bound integrins into the plasma membrane LR region [31]. KSHV induces the LR translocation of integrins to associate and to activate strictly LR associated entry receptor EphA2 resulting in enhancement of EphA2 kinase action that amplifies the downstream signals [52]. KSHV also simultaneously induces the LR translocation of calcium and integrin-binding protein-1 (CIB1) to aid in EphA2 initiated signal amplification. CIB1 sustains EphA2 phosphorylation and simultaneously associates with Src, c-Cbl, PI3K, alpha-actinin 4, and myosin IIA to enhance EphA2 crosstalk with the cytoskeleton to recruit macropinosome complex formation, thereby regulating productive KSHV trafficking towards the nucleus of infected HMVEC-d cells [74]. In contrast, NLR localized KSHV bound αVβ5 integrins are polyubiquitinated by c-Cbl and directed to the clathrin-mediated non‑infectious lysosomal pathway [52]. Therefore, LRs initiate the primary step of KSHV entry associated receptor‑signal complex segregation and mechanistic modulation towards KSHV macropinocytosis.

## 6. KSHV Entry Pathways

KSHV infects a variety of target cells* in vivo* and* in vitro*. KSHV genome and transcripts have been detected in CD19+ peripheral blood B cells, endothelial cells, monocytes, keratinocytes, and epithelial cells [82]. *In vitro* KSHV infects a variety of target cells, which include HMVEC-d cells, HUVEC cells, HFF cells, TIME (human endothelial cells immortalized by telomerase), HEK293, VERO, CV-1 (monkey kidney cells), and mouse fibroblasts [16,17,18,19,34,36,83]. The broad cellular tropism of KSHV is certainly due to its capability of utilizing different routes of entry depending on the cell type. KSHV enters human B cells, fibroblast, epithelial, and endothelial cells by endocytosis [37,47,66,84,85]. Electron microscopic studies provide evidence of KSHV internalization into irregularly cup shaped endocytic vesicles as early as 5 min post-infection in HMVEC-d and HFF cells [17,84]. Moreover, anti-gB and anti gpK8.1A antibodies can detect KSHV inside endocytic vesicles [17,65,66] and virus penetrates through the cytosol to reach near the nuclear periphery within 15 min as observed by virus capsid detection [66].

Cellular endocytosis involves four major routes: phagocytosis, macropinocytosis, clathrin-mediated endocytosis, and caveolae mediated endocytosis. Specific inhibitor studies have confirmed the cellular pathways hijacked by KSHV to gain access to different target cells. In endothelial cells (HMVEC-d and HUVEC), macropinocytosis provides a major route for the productive infection of KSHV. Studies with macropinocytosis inhibitors such as EIPA and rottlerin demonstrate a significant inhibition of both KSHV entry and gene expression [66]. Co-endocytosis experiments using macropinocytosis marker dextran with KSHV, and DiI-KSHV (envelope labeled virus) with Rab5 identify KSHV induced macropinocytosis events in both endothelial cell types by confocal and regular immunofluorescence microscopy, and also by flow cytometry [66]. This observation is strongly supported by control studies using clathrin pathway marker transferin and KSHV co-endocytosis experiments, which does not show any appreciable colocalization [66]. In contrast, another study claims clathrin-mediated endocytosis to be the predominant route of KSHV entry in endothelial cells [65]. This discrepancy is potentially due to the concentrations of inhibitors used and also the quantification method used to analyze KSHV entry.

Macropinocytosis involves active participation of the cellular cytoskeleton and formation of membrane ruffles, lamellipodia and blebs. Macropinocytosis of KSHV involves a membrane blebbing event in HMVEC-d and HUVEC cells (Figure 1). Treatment with blebbistatin, a potent inhibitor of membrane blebbing, inhibits KSHV entry significantly [54]. LRs play a critical role in receptor and signal clustering during HMVEC-d cell entry by KSHV [31]. Studies have deciphered the role of LRs for directing clustering of KSHV bound receptors EphA2 (strictly LR bound receptor in HMVEC-d cells) and integrins to direct macropinocytosis towards a successful latent infection and non-LR associated receptors towards a clathrin mediated non-infectious degradative pathway [31]. EphA2 initiates KSHV induced signal amplification and adaptor molecule CIB1 synergizes to sustain the feed forward amplification to promote macropinocytosis [74].

**Figure 2:**
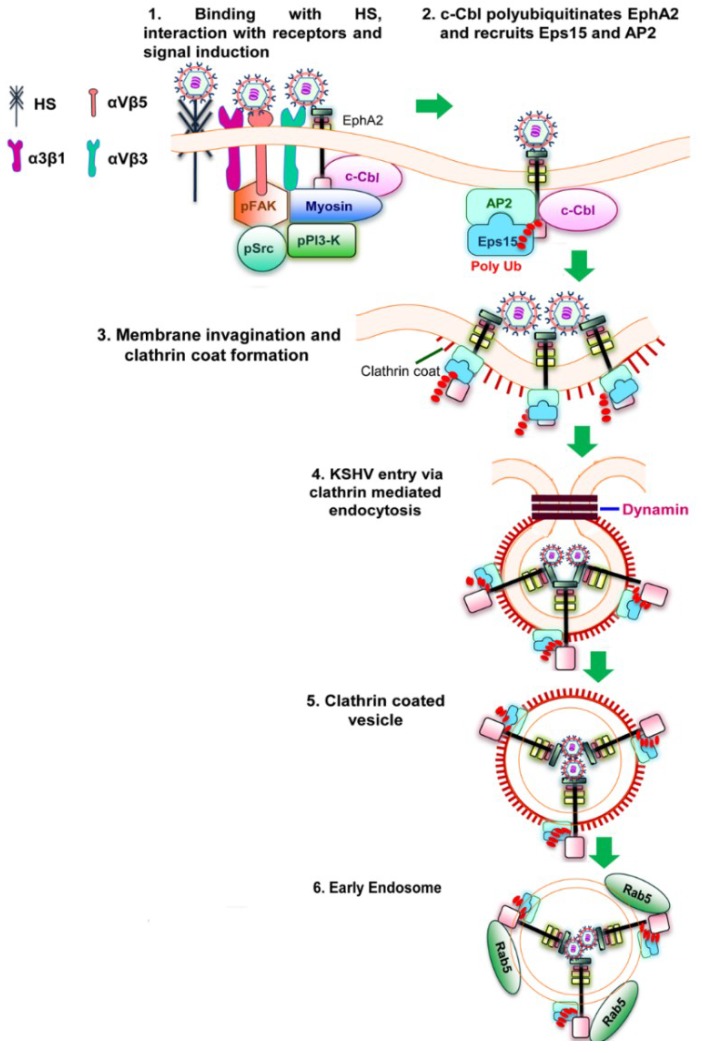
Schematic diagram showing clathrin mediated endocytosis of KSHV in HFF cells [51]. (**1**) KSHV glycoproteins bind and interact with heparan sulfate, α3β1, αVβ3 and αVβ5 integrins followed by their interaction with EphA2. The association of EphA2 with integrins leads to the formation of an active signaling complex among integrins, c-Cbl and myosin with simultaneous induction of FAK, Src, and PI3-K; (**2**) Activated c-Cbl polyubiquitinates EphA2 and recruits the accessory proteins Eps15 and adaptor protein AP-2 to mediate the endocytosis of the virus; (**3**) This is followed by the activation, recruitment and assembly of clathrin for the formation of clathrin coated pits; (**4** and **5**) The activated signaling platforms and the associated molecules leads to the internalization of KSHV into clathrin coated vesicles and dynamin dependent release of the vesicles; (**6**) The internalized vesicles also recruit Rab5 and transports KSHV to a productive infectious pathway and gene expression.

In HFF cells, KSHV enters via clathrin mediated endocytosis. Chlorpromazine, a clathrin pathway inhibitor significantly inhibits KSHV entry into HFF cells, whereas nystatin, an inhibitor of caveolae, and cholera toxin B, a LR inhibiting agent, have no effect on entry [66,84]. A recent study shows that interaction of KSHV with EphA2 and polyubiquitination of EphA2 by c-Cbl is essential for clathrin mediated endocytosis of KSHV in HFF cells. The mechanism of EphA2 dependent KSHV entry via clathrin mediated endocytosis include binding and interaction of KSHV with HFF cell surface heparan sulphate, integrins (α3β1, αVβ3 and αVβ5), and with EphA2 in the non-LR region. Interaction with EphA2 results in the formation of an active signaling complex among integrins, c-Cbl and myosin IIA with simultaneous activation of FAK, Src and PI3-K. Morever, c-Cbl polyubiquitinates EphA2 and recruits the accessory proteins Eps15 and adaptor protein AP-2, which promote the activation, recruitment and assembly of clathrin to the formation of clathrin coated pits (CCP). These signaling complex and associated events perform several functions including internalization of KSHV into clathrin coated vesicles, dynamin dependent release of endocytic vesicles and also the trafficking of KSHV into the Rab5 early endosome for successful infection [51] (Figure 2). In addition to HFF cells, KSHV utilizes clathrin mediated endocytosis to gain access to BJAB and HEK293 cells [37]. In the monocytic cell line THP-1, KSHV is known to enter by both clathrin and caveolin dependent endocytosis [35].

## 7. KSHV Trafficking

KSHV penetrates the host cell cytosol and delivers its genome to infected cell nuclei as early as 15 min post-infection [86], and delivery of KSHV DNA to the nucleus is maximal 90 min post-infection establishing KSHV trafficking as a very rapid process [57,58,66,68,87]. KSHV enters into HMVEC-d cells via macropinocytosis and in to HFF cells via clathrin mediated endocytosis followed by cytosolic penetration regulated by dynamic action of Rho-GTPases to utilize the microtubular network [57,88]. Inhibitor studies affecting Rho-GTPase upstream such as microtubule destabilizing agent, nocodazole, and PI3-K inhibitor significantly blocks KSHV nuclear entry but not binding and endocytosis [58,66,68]. KSHV actively utilizes cytoskeletal motility to promote nuclear trafficking where microtubule depolymerization inhibits infection in target fibroblasts and endothelial cells [57,68]. The critical role of Rho-GTPase has been studied by Rho activating *E. coli* cytotoxic necrotizing factor to show enhanced intracellular trafficking and nuclear delivery of KSHV genome whereas Rho inhibitory factor CdTxB treatment abolishing microtubular acetylation also inhibits KSHV nuclear delivery [68]. Subsequent steps for KSHV nuclear delivery are carried by dynein motor proteins, which govern ATP dependent retrograde transport (minus end transport involved is cargo trafficking from cell periphery towards the vicinity of the nucleus) along with the microtubule. Inhibiton of dynein motor proteins by sodium orthovanadate significantly abolishes the KSHV nuclear delivery process [68]. Hence, KSHV penetration through the host cytosolic barrier is a concerted event between host and cellular proteins leading towards a successful nuclear delivery of KSHV DNA.

In HMVEC-d cells, confocal microscopic studies reporting KSHV productive cargo internalized via EphA2 and CIB1 synergized macropinocytosis, demonstrate selective association of EphA2-KSHV-Rab5 and CIB1-KSHV-Rab5 in early macropinosomes by triple colocalization. Additionally, shRNA studies for the respective molecules significantly abolishes KSHV trafficking into Rab5 positive vesicles, strongly suggest the importance of macropinocytosis in establishing productive infection [74]. In HFF cells, shRNA studies for EphA2 also show significant reduction in KSHV sorting into Rab5 positive vesicles confirming the global requirements of KSHV trafficking pathways [51].

KSHV productive infection is orchestrated by several host transcription factors, playing regulatory roles during post entry stages to establish *de novo* KSHV infection. Mitogen activated protein kinase (MAPK) pathways and extra cellular-signal-regulated-kinase (ERK) pathways are activated by KSHV as early as 5 min p.i. [68]. In HFF and HMVEC-d cells, soluble KSHV gpK8.1A treatment can induce MAPK mediated ERK1/2 phosphorylation [89]. PI3-K and protein kinase C-zeta are reported as upstream in this pathway and have been validated by specific inhibitor studies with PI3-K, PKC-Zeta, MEK, and ERK to reduce KSHV infectivity without affecting its binding to the target cells. In addition to ERK, KSHV also induces crucial transcription factor NF-κB as early as 5–15 min p.i., followed by nuclear translocation of p65-NFkB [90]. Infection with heparin treated KSHV and pretreatment of target cells with Bay11-7082 is a drastic reducing factor for NF-κB activation [90]. Inhibition of NF-κB also greatly reduces ORF50 and ORF73 gene expression without affecting entry inhibition. Inhibitor studies provide evidence that activation of NF-κB leads to the activation of several host transcription factors such as Jun D, Jun B, phospho-c-Jun, cFos, and FosB factors which probably also contribute towards latent infection.

## 8. Conclusions

The mechanism of KSHV cell entry involves a sequence of events which include interaction with receptor, endocytosis, trafficking, and nuclear delivery. Receptors are the critical molecules in target cell recognition, and multimeric receptor complex formed at the membrane link the virus to the intracellular tyrosine kinases, cytoskeletal proteins, GTPases, and adaptor molecules to facilitate the entry of the virus into the cytosol. Current research has discovered that macropinocytosis and clathrin‑mediated endocytosis are the major routes of entry in the natural target cells of KSHV. The data from receptor and entry inhibitor studies provide significant information for the design of future drugs that can efficiently block the entry of the virus. Although these studies have provided a wealth of knowledge on KSHV entry, more extensive research is required to understand the additional cytosolic factors that determine rapid endocytosis and the protein complexes that are involved at each stage of infection. Future studies are also needed to understand the molecular and biological characteristics of the host immune modulators and viral proteins required for the virus to escape from host immune detection during entry and nuclear delivery of viral genome.
